# 

**Published:** 1923-06

**Authors:** 


**THE DENTAL REGISTER** is published every month by the Samuel A. Crocker Company, 18 W. 7th Street, Cincinnati, O. It is mailed to subscribers during the last week in each month, being a month-end publication. Have copy for current month here by the 15th of each month. Copy received by the 15th of the month goes into that month's number of the Dental Register. Publication and business office, 18 W. 7th Street, Cincinnati, Ohio. Editorial office, 18 W. 7th Street, Cincinnati, Ohio. Address all correspondence to Editor Dental Register, Box 838, Cincinnati, Ohio, c'o The Samuel A. Crocker Company.

Notices of meetings, announcements of interest to the dental profession, and particulars of new ideas and new inventions will be given free publication in reading section. Advertising section is open to all ethical advertisements.

We are not responsible for opinions or statements made by writers whose articles this magazine may publish—nor does the publishing of any article infer in any way that we indorse the views or opinions expressed by any writer whose article we may publish.

### Sal Hepatica

Materially Aids
Local Treatment
IN
PYORRHEA

Samples on Request

Bristol - Myers Co.
NEW YORK

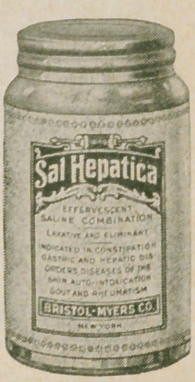

### DIRECTORY

FOR

Members of the Dental Profession whose practices are limited to a specialty.

The object of this directory is to furnish ready reference to specialists who take referred patients. If you wish your name inserted in this list please correspond with the Dental Register, Box 838, Cincinnati, Ohio.

Your name, specialty, office hours and phone number will be published.

This entire page is reserved for Professional Directory.

### Cincinnati

Dental X-Ray Diagnosis
CHARLES K. FIELD
2709 Woodburn Ave., Walnut Hills
Hours, 10 to 4
Woodburn 2486

Periodontoclasia (Pyorrhea and Oral Prophylaxis)
EDGAR H. EBERLE
605-06 Union Central Bldg., 4th and Vine Sts.
Hours, 9 to 5—Consultation hours by appointment
Main 882

Periodontoclasia (Pyorrhea and Oral Prophylaxis)
MARIE L. BECKERT
25 Groton Bldg., 7th and Race Sts.
Hours, 9 to 5—Consultation hours by appointment
Canal 842

Exodontia (Extraction and Oral Surgery)
ROBERT M. SCHELL
20 W. 9th St., between Vine and Race Sts.
Hours, 1 to 5—Consultation by appointment
Canal 118

### DENTAL REGISTER

*ON TO CLEVELAND, SEPTEMBER 10-14*

### EXPECT 10,000 DENTISTS TO ATTEND CONVENTION

*Members of Committee Begin Preparations for Meeting of Association Here Next Fall*

Every hotel in the city is being canvassed in an effort to find rooming space for between 8,000 and 10,000 dentists and about 5,000 members of auxiliary professions and businesses who will invade Cleveland for the annual meeting of the American Dental Association, September 10 to September 14.

Dentists from Europe, as well as Canada and the United States, are expected for the sessions.

Five hundred clinics have been arranged at hospitals, hotels and public hall, where the main sessions will be held. The local committee on arrangements, numbering 100 Cleveland dentists, met at The Hollenden to discuss plans for the convention.

A meeting of officers of the American Dental Association, held in Cleveland recently to look over arrangements, said the plans made by Cleveland were the best that ever have preceded a meeting of the association.

John P. Buckley is president of the American association. Besides the main sessions there will be many section meetings and conventions of auxiliary bodies and supply dealers.—*Cleveland Plain Dealer.*

---

### EXHIBIT COMMITTEE

The Exhibit Committee of the A. D. A. wish to place before the members of the A. D. A. the fact that the Dental Exhibit at the meeting of the A. D. A. at Cleveland, Sept. 10th to 14th inclusive, will be the largest, most elaborate and instructive that has ever been held in connection with the A. D. A. We want you to realize that the Dental Exhibit will not be simply a side issue or event of the meeting but will be one of the main attractions of the meeting of the A. D. A.

In order to firmly impress this upon your mind it will be only necessary to state a few facts, namely:

The Exhibit Hall at the Public Hall is the most complete hall for this purpose in America. It is well lighted. The ventilation is superior to any other hall in America. Every convenience is already installed for the use of the exhibitor. Every arrangement has been made for utility and service, and it is the aim of this committee to give a service to the exhibitor he has never enjoyed before. This committee have these arrangements practically completed.

The members of this committee want the members of the A. D. A. to understand that we appreciate fully the immense value of a complete and diversified exhibit and we aim to give them something that they can not afford to miss.

As far as the exhibits are concerned the success of this meeting is already assured, because we have signed contracts for more exhibits than any ever held in conjunction with any meeting of the A. D. A.

The sessions, registration, etc., of the meeting has been so arranged that eighty per cent. (80%) of the activities of the meeting will be held in the Public Hall, where the exhibits will also be held.

In the face of these facts—how can you afford to miss this meeting? You certainly can not.

Come to the Cleveland meeting. Boost the Cleveland meeting and help yourself and your neighbor.

---

### HOW TO REACH CLEVELAND

Plan your vacation so you can attend the meeting of the A. D. A. in Cleveland. You will enjoy cool, invigorating September weather after August's heat.

Cleveland, situated on Lake Erie, is one of the main ports on the greatest chain of inland lakes in the world.

Men from the West can arrange their itinerary by way of Duluth, thence across Lake Superior, through the great locks at the Soo, down St. Mary's River and Lake Huron, Lake St. Clair, beautiful St. Clair flats and Detroit River to Lake Erie.

Stop-overs can be arranged along this inland water route which will enable you to see some of the most beautiful scenery North America has to offer, Macinac Islands and Georgian Bay.

Those coming from New York and other points East, can travel all the way to Cleveland by water, with only three short connecting links by rail. This route includes Hudson River, passing the Palisades and Catskills to Albany, north to picturesque Lake George and Lake Champlain, nestling in the heart of the Adirondack Mountains, affording scenery which is not surpassed elsewhere in the United States.

From Lake Champlain, a short jump by rail brings the traveler to Montreal, a city of many interesting sights.

From Montreal, the trip down the wonderful St. Lawrence River past the Thousand Islands, brings to you scenery never to be forgotten. Then across Lake Ontario, touching Toronto and Buffalo with stop-overs along the route, including Niagara Falls, which we all know as the largest waterfall in the world.

From Buffalo we approach Cleveland via Lake Erie.

No one can know the natural wonders of their country without having seen this chain of beautiful waterways.

From Cleveland numerous short side trips by water can also be arranged. Cleveland is readily accessible by rail from East, West and South.

Members and their families wishing to tour to Cleveland will find splendid roads leading there from East, West and South.

---

REGISTRATION AT THE MEETING

The registration for the Cleveland meeting will be handled in a newer, better and more efficient manner than any previous meeting. Experts on this problem have been consulted and a plan has been formed whereby much of the confusion and delay usually accompanying the registration of eight to ten thousand people has been eliminated.

Each visitor will be given a card made out for him. These cards will be handled by experts who will be able to give out information very rapidly. The facilities for registration at the hall are ideal and very little of the time of the members will be taken in the task of registration.

This department will provide a daily list of all registered and have it classified by states.

This list will be published and also provided for the use of the Information Committee. It will then be a simple matter to find out if a friend whom you have not seen for years is in attendance or not.

---

### ILLUSTRATED CLINICS

Every member of the Committee on Illustrated Clinics has pledged himself to give his utmost attention to the needs of the essayist or clinician requiring the use of a lantern to illustrate his subject.

To facilitate the efficient distribution of the lanterns, a large chart is being prepared showing the number of lanterns in use, when and where being used. This chart, which will hang at headquarters, Public Auditorium, will show at a glance where the lanterns are situated. It will also show the name and hotel room number of the essayist or clinician using a lantern.

A member of this committee will be in charge at each section's headquarters to see that the lantern is ready when needed. He will also see that the slides are properly received and returned to the essayist without breakage or loss of time, thus making it possible to perform our share in making this Cleveland meeting a big success.

---

### MOUTH HYGIENE OR SCHOOL DENTISTRY

If you are interested in Mouth Hygiene or School Dentistry, come to Cleveland, September 10th to 14th. An excellent exhibit relating to school dentistry will be here for your study. Cleveland is one of the historic centers of the Mouth Hygiene movement. In Cleveland the Marion School experiments were conducted in 1910-11.

In Cleveland the Board of Education has conducted Mouth Hygiene Dispensaries in the school buildings for seven years. In Cleveland, Mouth Hygiene work has been carried on in Municipal Health Centers for six years. In Cleveland a Surgical Dental Dispensary has been conducted at City Hospital for patients sent from our Mouth Hygiene Dispensaries. In Cleveland the Tuberculosis Sanitarium has had full time dental service for six years. Cleveland has supported an active Mouth Hygiene organization for eleven years. Let us show you our clinic work, and let us tell you of the great change in the number of children receiving dental care in the last ten years. Let us tell you how we finance the service and let us show you how we conduct a Mouth Hygiene Dispensary.

---

### CLINICS

The Clinic program will be the largest and most elaborate ever presented at a national meeting. There will be *Individual Clinics* to satisfy the man who likes the old-fashioned personal contact and conversation type of clinic.

There will be *Group Clinics* in which each man will, with great pains, demonstrate a particular step in technic. This type of clinic is decidedly educational, presenting the most approved methods in technic in an ideal manner. There will be *Surgical Clinics* for those interested in operative procedure under anaesthesia. Every technical procedure in dentistry will be represented on the Clinic program. Two half days of Clinic program, Thursday afternoon and Friday morning, will give ample opportunity for all in attendance. The Clinics will be so arranged that each half day will present half of the group of clinics on each subject. Some of the clinics will be designed to demonstrate the papers presented in the sections. Eighty-five per cent. of all knowledge is acquired through the eyes; therefore, come and enjoy the Clinic program and carry home a fund of ideas that will be helpful in practice.

The Clinic program, with the exception of Surgical Clinics, will be held in the six million dollar ($6,000,000) auditorium. The most wonderful lighting system known is in this building. Every dentist will enjoy the play of colors that flood the auditorium on appropriate occasions. This great auditorium is delightfully located near the banks of Lake Erie, some 70 feet above the water level, overlooking a most interesting harbor where iron ore, limestone and coal meet. This auditorium is a part of the famous mall which Cleveland has been developing during the past two decades.

Cleveland is far enough west to appreciate western hospitality, and we assure you a happy and interesting week—September 10 to 14, 1923.

---

DENTISTRY IN THE CLEVELAND HOSPITALS

Some of the visitors to the American Dental Association Convention will be interested in seeing some of the work being done in Cleveland hospitals. Cleveland City Hospital has a well organized Dental Staff. At this hospital, which is a municipal institution of over 1,200 beds, the Dental Staff is responsible for a complete department and is recognized as of equal grade and standing with the departments of Surgery, Medicine, etc. The staff consists of a department head, two or more visiting dental surgeons and a resident dental surgeon. Including the outpatient dispensary cases this department renders services to 4,000 to 5,000 patients per year. Most of these require general anaesthetics. Also Nitrous Oxide—oxygen anaesthetics for surgical operations outside this department are usually administered by a member of the Dental Staff. Operations in this department include everything in the catalogue of jaw surgery, but consist mostly of exodontia cases.

The Dental Department is also responsible for a teaching course on Dental Surgery and Mouth Hygiene in the

School for Nurses which is attached to the hospital. This is a required course and student nurses must attain a passing grade before they are eligible for graduation.

Besides seeing strictly dental cases the Visiting Staff works constantly in co-operation with staff men of other departments in matters of diagnosis and treatment of hospital cases.

Dr. Herman C. Kenyon inaugurated this service six years ago and has been the head of the Dental Staff since that time. The other members of the staff at this date are Drs. P. J. Aufderheide and W. A. Nichols.

Visiting members of the American Dental Association will be welcome at the City Hospital.

---

MOUTH HYGIENE AND PUBLIC INSTRUCTIONS

Last year President Hartzell requested the Council on Mouth Hygiene and Public Instruction of the American Dental Association to prepare a Health Exhibit for the Los Angeles meeting. Many organizations were represented and the exhibit far exceeded the expectations of the Council both in the number of exhibits and the interest shown in the exhibition. In fact, so great interest was shown that Dr. Buckley notified the Council to stage another Health Exhibit at the Cleveland meeting.

The Local Committee thought so favorable of the Health Exhibit that they gave us the best space in Cleveland, the front of the main floor of our new auditorium building. Fourteen thousand square feet have been alloted and possibly more will be requested. The Health Exhibit will cover all scopes of educational dentistry. Everyone interested in educating the public should plan to spend considerable time at the exhibit.

If you want to start a Dental Clinic in your schools, settlement houses, Y. M. C. A.'s and factories, you can learn all the details and many other interesting facts at this exhibit.

The list of contributors that have already been provided space to exhibit include societies from the following states:

Massachusetts, New York, Ohio, Pennsylvania, West Virginia, Indiana, Michigan, Illinois, Wisconsin, Minnesota, California, Mississippi, North Carolina and Louisiana.

We also expect exhibits from the Forsythe and Eastman Dental Dispensaries, the McDowell County, W. Va., Clinics, City of Cincinnati, The Child Health Demonstration of the National Health Council, the National Association of Industrial Dental Surgeons, The National Cash Register Company, Metropolitan Life Insurance Company, The National Lamp Company, Cleveland Mouth Hygiene Association and about ten local child helping organizations that are willing and anxious to exhibit, because of their appreciation of the value of Mouth Hygiene in the work they are doing. Dr. Weston A. Price will have a very interesting exhibit of work on Dental Research as will also the Dental Hygienists of California.

The Committee on Information will have booths and attendants at the Public Hall, and at all of the down-town hotels. Our work will be greatly assisted by the daily news which will be issued each day of the convention. Members of the committee will be prepared to give out information continuously and we hope to be able to answer every inquiry.

---

### "NON MULTA, SED MULTUM"

We all know more because each one of us is not expected to know so many things.

The whole world is divided into two sections. One stirs around and does things, and t'other stands by and just naturally wants to know why in thunder they don't do 'em different.

Which is your section?

### A WELCOME TO CLEVELAND

The importance of an association like the American Dental Association, which has as one of its objects the gathering of exhaustive and scientific research which but few men ordinarily are able to engage in, and through its members to add largely to the store of general knowledge by disseminating the results of their special and expert labors, can not be over-estimated.

What the busy practitioner needs is that someone should, to an extent, do various specialized work for him. It is absolutely necessary that he should get the concrete results of the labors of others, and that those results should be placed before him in an attractive and easily assimilated form. To accomplish this the officers and committeemen have been laboring continuously for many months.

One can not be expected to assimilate all that the completed program offers. The world has profited enormously by the tendency of men to confine their attention to particular subjects and to thoroughly master them.

Make your plans for the summer to include a visit to the Cleveland meeting and we will help you enjoy the specialized labors of others in that field most attractive to you.

On behalf of our Hospitality Committee, I am extending to you a most earnest and cordial welcome.

It is also expected that you notify us of your time of arrival and route of entry. We are at your service from the time of arrival until after the meeting as long as you can stay with us. Notices may be sent to J. F. Stephan, Chairman, Hospitality Committee, Hollenden Hotel.

---

### ENTERTAINMENT COMMITTEE, AMERICAN DENTAL ASSOCIATION

The Entertainment Committee, in behalf of the dentists of Cleveland and Ohio, are preparing a cordial welcome for our thousands of visitors who will be here when the members and ladies of the American Dental Association come to the convention September 10th.

All are enthusiastic in their desire to extend the famed hospitality of the Old Western Reserve and so a program is being perfected which will keep all busy playing when not working.

Our first desire is to have every visitor see our wonderful city and its beautiful homes. Cleveland is reputed to have a greater percentage of home owners in proportion to the population than any other city in the country. Many of its fine residence districts resemble parks. We also have one of the finest park systems to be found in any city in the country. Cleveland is built on the shores of Lake Erie, one of the great inland seas, and Cleveland is one of the greatest ports in the world, when calculated by the number of tons of cargo unloaded from ships. It is one of the great ports for receiving iron ore and shipping coal. It is famed for its manufactures and comprises thousands of factories producing every conceivable article of commerce from clothing to suspension bridges.

Unique entertainment features have been planned, but the greatest treat in store will be the "Dentists' Night" at Keith's Palace Theatre, the most handsome and best equipped theatre in the world. The entire theatre has been chartered for the American Dental Association for Thursday evening of the convention week and the dentists will own and operate this wonderful house from cellar to garret on that occasion. The entire vaudeville show, especially arranged for us, will be ours, as well as every other feature of the theatre. Every seat in the house, three thousand eight hundred (3,800) in all, will be occupied by dentists and their ladies.

Automobile and sight-seeing trips have been arranged and a special expedition for the ladies is being perfected; we will keep this a secret, but the ladies will remember Cleveland and this trip for many a day.

The opening of every general session will be featured by a brief but snappy entertainment number, to put all in good humor and in a receptive mood for the scientific program to follow.

The wonderful organ in the Cleveland Auditorium will be played by a Cleveland dentist and other Cleveland dentists will contribute their parts to these entertainments.

There are many of the finest restaurants and cabarets in the country to be found in Cleveland and so our guests can look forward to much work at the convention and much pleasure during their leisure.

The motto of the Entertainment Committee is: "A little folly now and then is relished by the wisest men."

---

SUGGESTIONS FROM THE HALLS AND HOTELS COMMITTEE

Hotel rates will not be advanced.

All hotels are within ten minutes walk of the auditorium. There is practically no choice in the large hotels as to location or service. Everyone should read the Reservation Blank carefully and do as directed. These blanks have been published in the Journals.

A large number of reservations have been made and the indications are that the attendance will surpass all other meetings.

To handle the big crowd co-operation is necessary. It is earnestly requested that as far as possible when two or more can occupy a room they should do so.

***ON TO CLEVELAND, SEPTEMBER 10-14***

### AMERICAN DENTAL ASSOCIATION

### TWENTY-SEVENTH ANNUAL MEETING

### Cleveland, O., September 10-14, 1923

### HOTEL RESERVATIONS

In securing hotel reservations for the coming convention, consult the hotel rate-sheet and fill out the blank application below. Mail it *immediately* to the *hotel* you wish to patronize. The hotel will then advise you of the reservation which they make for you.

S. MARSHALL WEAVER, Chairman, Hotels and Halls Committee,
618 Rose Building, Cleveland, Ohio

**KEEP THIS REMINDER OF YOUR RESERVATION**

Hotel Selected ..........................................

No. of Rooms ..........................................

No. of Persons ..........................................

Date of Reservation ..............................1923.

Detach here and keep the above for reference

---

### MAIL THIS APPLICATION DIRECT TO HOTEL

**HOTEL RESERVATION**

**American Dental Association, Cleveland, O., Sept. 10-14, 1923**

.....................Hotel ....................1923

Cleveland, Ohio

**Please reserve the sleeping accommodations noted below:**

......Room(s) with bath for......people. Rate desired $......
(per day)

......Room(s) without bath for......people. Rate desired $......
(per day)

I hereby agree to pay for rooms described from morning/evening of September............ to September............, unless cancellation is made by August 20, 1923.

**List names and addresses of all who will occupy the room with you.**

Name.......................... Address........................
Street, City and State

Name.......................... Address........................
Street, City and State

Name.......................... Address........................
Street, City and State

Name ..........................................

Street ..........................................

City .......................... State................

### HOTEL ROOM RATES—CLEVELAND

| HOTEL | Room Capacity | Room With Bath: One person per day | Room With Bath: Two persons per day | Room Without Bath: One person per day | Room Without Bath: Two persons per day |
|---|---|---|---|---|---|
| ANSONIA<br>83848 Prospect Ave. | 76 | 2.00-2.50 | 3.50- 4.50 | | |
| CLARENDON<br>St. Clair Ave. & Ontario | 50 | | | 1.25-1.50 | 2.00-2.50 |
| CLEVELAND<br>Superior Ave., Public Sq. | 1000 | 3.00-7.00 | 4.50-10.00 | | |
| COLONIAL<br>523 Prospect Ave.. S. E. | 150 | 2.50-3.50 | 4.00- 5.00 | 2.00-2.50 | 3.00-3.50 |
| DOANBROOKE (Apts.)<br>E. 105th at Euclid Ave. | 122 | 2.50-7.50 | 4.00-10.00 | | |
| EUCLID<br>Euclid Ave. at E. 14th St. | 200 | 2.50-3.50 | 4.00- 6.00 | 1.50-3.00 | 2.50-4.00 |
| GILLSY<br>1811 E. 9th St. | 350 | 2.00- | 3.00- 4.00 | 1.50- | 2.50-3.00 |
| HOLLENDEN<br>Superior Ave. at E. 6th St. | 800 | 3.00-6.00 | 5.00- 8.00 | | |
| MECCA<br>1862 E. 9th St. | 120 | 1.50-2.00 | 3.00- | 1.50- | |
| NEW AMSTERDAM<br>Euclid Ave. at E. 22nd St. | 350 | 2.00- | 3.50- 4.00 | | |
| OLMSTED<br>Superior Ave. at E. 9th St. | 300 | 2.00-4.00 | 4.00- 6.00 | | |
| STATLER<br>Euclid Ave. at E. 14th St. | 1000 | 3.00-8.00 | 4.50-10.00 | | |
| TALGARTH<br>1924 Prospect Ave. | 75 | 1.50-2.50 | 2.00- 3.50 | 1.25-1.50 | 1.75-2.25 |
| WINTON<br>Prospect Ave. at E. 9th St. | 600 | 3.00-5.00 | 5.00- 8.00 | | |

That all members desiring to attend the Contention may be comfortably housed in hotels in the downtown district, the Hotels of Cleveland have been asked to fill each room to its regular capacity. Members, therefore, are requested to procure a room-made or mates wherever possible, and request room for goups of 2, 3 o 4 persons.

### GENERAL INFORMATION

### Clubs

Cleveland Athletic Club, 1120 Euclid Ave.
Union Club, 1211 Euclid Ave.
University Club, 3813 Euclid Ave.
Y. M. C. A., 2200 Prospect Ave.
Y. W. C. A., Prospect Ave. and East 18th St.

Sections have been assigned to individual hotels where all their meetings will be held.

All Headquarters are within five minutes walk of each other.

SECTION HEADQUARTERS

General Sessions, Public Auditorium.
Operative Dentistry, Cleveland Hotel.
Full Denture Prosthesis, Winton Hotel.
Partial Denture Prosthesis, Winton Hotel.
Oral Surgery, Hollenden Hotel.
Orthodontia, Statler Hotel.
Peridontia, Statler Hotel.
Scientific Research, Public Auditorium.
Mouth Hygiene and Public Instruction, Public Auditorium (main floor).
Clinics (except surgical), Public Auditorium.
Exhibit, Public Auditorium.

HOUSING HEADQUARTERS

Official Family, Hollenden Hotel.
Delta Sigma Delta Frat., Statler Hotel.
Psi Omega Frat., Cleveland Hotel.
Xi Psi Phi Frat., Winton Hotel.
Alpha Betta Gamma Frat., Winton Hotel.
Sigma Epsilon Delta Frat., Winton Hotel.

---

SPECIAL NOTICE ABOUT TRANSPORTATION TO CLEVELAND

Those attending the meeting and wishing to make the trip one way or round trip to Cleveland by water via the Great Lakes from the West or the St. Lawrence River from the East route, should get in touch with the nearest steamship agent or apply to Dr. W. J. Pryor, Local Arrangements Committee, 761 Rose Building, Cleveland, Ohio, for information regarding same before purchasing railway tickets.

---

A REPORT FROM THE GENERAL CHAIRMAN, LOCAL ARRANGEMENTS COMMITTEE

On behalf of the Local Arrangements Committee, I wish to extend a cordial welcome to the members of the American Dental Association to come to Cleveland in September to attend what we believe will be the most profitable and largest dental meeting ever held.

In making this statement we do not wish to cast reflections upon any previous meeting, but realize that in the natural evolution of the progress of Dentistry, if such were not the case, we would feel derelict in our duties and responsibilities. In all probability and by the natural trend of the times, the 1924 meeting should surpass this one. No doubt, each meeting marks a distinct epoch in the history of Dentistry.

This year the Local Arrangements Committee was enlarged from eight members to fifteen. The members of the committee were all selected by the Cleveland Dental Society, recommended to the president and trustees of the American Dental Association and by them officially appointed. The members were chosen for their peculiar fitness to the place where they were to serve and the work thus far has proven the feasibility of such a plan. Every man has displayed unusual ability and efficiency and we believe the American Dental Association will have the best managed meeting in its history. Practically all the important arrangements for the meeting have been completed.

The new Public Auditorium has been secured for the principal place of meeting, where it is intended to hold approximately eighty per cent. of the activities. The hall for the dental exhibits will be ideal and will accommodate a larger number than ever before. The character of the exhibits will be of the best and promises to be of great educational value.

Fortunately the location of the large hotels is most advantageous. They are grouped in the down-town district and within ten minutes' walk of the Public Auditorium. The Cleveland Hotel Men's Association have co-operated with us to the fullest extent. There will be no advance in hotel rates. They have agreed to place at our disposal sixty per cent. of the room capacity, which will make available approximately forty-five hundred rooms in the down-town district. Add to the number the available rooms in the outlying districts and it will bring the total to approximately sixty-five hundred rooms. This, we believe, will furnish accommodations for all in attendance, although it will be necessary in most instances for two or more people to occupy the same room.

The hotels have further agreed to place at our disposal without cost all halls and meeting rooms. The Hollenden agreeing, where headquarters of the meeting will be established, to give over the entire mezzanine floor.

Definite arrangements have been made for all meetings in so far as such arrangements have been requested. A master chart is in use which will show definitely the time and place for each and every meeting; when this is completed all confusion in regard to meeting places will be eliminated.

A folder containing a reservation blank and definite information regarding the hotels, such as rates, headquarters for various sections, and a diagram showing locations of the various hotels with respect to the Public Auditorium, was printed by the Hotel Men's Association and placed in the hands of the committee for distribution. These have been widely distributed through the Publicity Committee and will be copied and printed in several of the dental journals. The committee will co-operate with the hotels in an endeavor to keep all reservations straightened out so that there will be no confusion or misunderstandings in this respect.

The Entertainment Committee has practically completed its program which will include such entertainment as is customary for the general sessions, for the guests, for the ladies in attendance and for the general membership. The latter will be given in Keith's Palace Theatre, reputed to be the finest theatre in the world, on Thursday evening, September 13th. This entertainment promises to be quite an unique affair, combining a scientific program as well as an interesting and enjoyable time. The theatre will accommodate thirty-eight hundred. It will therefore be advisable for those who wish to attend this occasion to secure tickets upon arrival.

The Reception Committee will function during the entire meeting, will welcome guests, friends and members to Cleveland and will attempt to see that everybody is happy, contented and well satisfied.

The Information Committee will inaugurate a very definite and complete program and will endeavor to supply information pertaining to the meeting. Booths will be established at the Public Auditorium and at each of the large hotels which will make it convenient to secure any and all desired information.

The Committees on Transportation, Registration, Program Clinics and Illustrated Clinics have all formulated their policies and we are assured that all of the work concerning these committees will be most efficiently performed.

The Publicity Committee has been very active; have endeavored to secure all information of interest regarding the meeting and to disseminate the same through various sources. The Publicity Campaign was inaugurated several months ago and has been consistently carried out since then. This committee was active in developing the seal which carries the slogan of the meeting, namely, "Prevent," "Conserve," "Progress." This we believe to be the most appropriate and one which needs no apology. The committee also designed the beautiful cover page which is being used for the special Cleveland Number of the American Dental Association Journal.

The badges and membership buttons to be used at the meeting will be rather an unique feature. There will be a special badge which will have a name-plate, with a ribbon, on which will be suspended a pendant. These badges will be distributed to the officers, all committees, delegates and guests, the color of the ribbon and printing upon same indicating the respective office, committee, etc.

The membership button will be of celluloid, 1¼ inches in diameter, and a duplicate of the inside circle of the original seal, the slogan being omitted.

The visitor's badge will be the same design, of a different color and will have a small ribbon, on which will be printed ''visitor''.

The exhibitor's button will be of the same design, of still another color, and will have attached a ribbon on which will be printed the word ''exhibitor''.

For the Ohio members of the A. D. A. the regular membership button will be used with a ribbon attached on which will be printed ''Ohio''.

The color of the ribbon on the badge worn by members of the Cleveland committee, of which there will be more than one hundred, will be white, with the word ''Committee'' across the center. This will be the only white ribbon, therefore the Cleveland men will be easily distinguished.

Another special feature to be inaugurated will be the publication of a daily bulletin. This will be rather elaborate and will contain many things of interest to the members in attendance. There will be cartoons, current events, registration, information, and various other things. The first copy of this bulletin will probably be mailed to each member of the association two or three weeks previous to the meeting and about ten thousand copies printed each day of the meeting, beginning Monday and concluding with Friday's issue.

In conclusion, may I again call your attention to the slogan ''Prevent,'' ''Conserve,'' ''Progress,'' and offer the suggestion that in the light of the present knowledge and progress in Dentistry, no member can afford to miss this great and epoch-making meeting.

DR. F. M. CASTO,
*General Chairman.*

*ON TO CLEVELAND, SEPTEMBER 10-14*

AMERICAN DENTAL ASSOCIATION

First meeting of the American Dental Association, Washington, D. C., 1860.

In 1896, at Old Point Comfort, the American and Southern coalesced and adopted the name of National Dental Association.

In 1922, at Los Angeles, the name was changed to American Dental Association.

September, 1923, will again be the first meeting of the American Dental Association to be held at Cleveland, September 10-14.

*ON TO CLEVELAND, SEPTEMBER 10-14*

### ANNOUNCEMENTS

### THE AMERICAN DENTAL TRAPSHOOTERS' ASSOCIATION FIRST ANNUAL TOURNAMENT

Cleveland, Ohio, Sept. 10, 1923

The trapshooters of the American Dental Association will be interested to know that the grounds of the Northern Ohio Gun Club, at the foot of 49th Street, Cleveland, Ohio, will be open for practice Sunday, September 9, 1923, at 1 o'clock P. M. for all who wish to practice before the tournament which will start at 1 o'clock Monday, September 10, 1923.

The tournament will consist of four 25 target events, 16 yards rise. Each 25 target event will be a race and suitable trophies will be awarded to the high scores in each 25 target event. A championship cup will be awarded to the contestant making the highest score on the total of 100 target (four 25 target events). Ties will be shot off immediately after the close of the 100 target event.

The winner of the championship cup will not be eligible to win any of the trophies in the 25 target events. Should the contestant so desire, an Optional Sweepstake will be arranged at the time of making entries.

A good assortment of loaded shells will be for sale on the grounds at regular prices.

Through the courtesy of the Dupont Powder Company and the Winchester Repeating Arms Company, Mr. L. J. Squire and Mr. Jno. R. Taylor will manage this tournament.

### AMERICAN SOCIETY OF ORAL SURGEONS AND EXODONTIA

The annual convention of the American Society of Oral Surgeons and Exodontists will be held in Cleveland during the week preceding the American Dental Association meeting. Headquarters will be in the Hollenden Hotel. The hotel management have given every possible assistance to making this meeting a success and have reserved for our exclusive use all rooms necessary on the mezzanine floor.

The various committees have been hard at work and reports so far submitted go to show that one of the most successful meetings in the history of the organization will result.

The Program Committee and the Clinic Committee have reported the perfection of a most complete scientific program, and the Local Arrangements Committee have already perfected nearly all details for the meeting.

The Entertainment Committee have arranged to keep all happy during any leisure hours.

A dinner dance will take the place of the usual banquet, and other features are held in reserve as a surprise.

Elsewhere will be found the reports of other officers giving other details of this meeting.

The Ohio Society of Oral Surgeons extends a cordial welcome to all members of the American Society of Oral Surgeons and Exodontists. They hope for the pleasure of being our hosts.

